# Giant cementoblastoma involving multiple teeth: A rare case report and review of differential diagnoses

**DOI:** 10.1016/j.radcr.2024.02.026

**Published:** 2024-03-07

**Authors:** Dwi Noviyanti, Atta Kuntara, Eka Marwansyah Oli'i, Hasrayati Agustina

**Affiliations:** a Department of Radiology, Faculty of Medicine, University of Padjadjaran, Bandung City, West Java 40161, Indonesia; b Department Oral and Maxillofacial Surgery, Faculty of Medicine, University of Padjadjaran, Bandung City, West Java 40161, Indonesia; c Department of Pathological Anatomy, Faculty of Medicine, University of Padjadjaran, Dr. Hasan Sadikin General Hospital, Bandung City, West Java 40161, Indonesia

**Keywords:** Odontogenic tumors, Cementoblastoma, Mandible, Periapical radiopaque lesions

## Abstract

Cementoblastoma, a rare odontogenic benign tumor characterized by calcified cementum-like deposits produced by cementoblasts fused with the tooth root, represents a minute proportion of all odontogenic tumors, with a prevalence ranging from less than 1% to 6.2%. We present a case of a 19-year-old female experiencing pain, progressive swelling, and facial asymmetry in the left lower region over the 1 year ago. Physical examination revealed diffuse swelling in the left mandibular area, demonstrating tenderness and bony hardness on palpation. Radiographic findings displayed a giant, well-defined, relatively round, radiopaque mass, partially delineated by a thin radiolucent halo, and adhered to the roots of the teeth. Computed Tomography imaging revealed an osteoblastic hyperdense mass with extensive buccal and lingual extension. Sagittal images illustrated the mass's continuity with the root apex of the first molar, accompanied by a well-defined hypodense border. A biopsy confirmed the diagnosis of cementoblastoma, leading to the patient undergoing a left hemimandibulectomy. Given the various periapical radiopaque lesions that serve as potential differential diagnoses for cementoblastoma, the ability of the radiologist to distinguish their imaging characteristics plays a crucial role in determining an accurate diagnosis.

## Introduction

Cementoblastoma is a rare odontogenic benign tumor characterized by calcified cementum-like deposits produced by cementoblasts that fused with the tooth root [1,2]. It represents a minute proportion of all odontogenic tumors, with a prevalence ranging from less than 1% to 6.2% [3], [4], [5]. First recognized by Dewey in 1927, its prevalence is higher in children and young adults under 30 years of age [5] with a slightly higher incidence in males than females. The mandible is the preferred site over the maxilla [3,6] and the lesion is typically observed in the posterior region of the mandible, frequently involving a first molar. Lesions can vary in size, ranging from a small 0.5 cm lesion to a large mass measuring up to 5.5 cm [1].

Differential diagnoses for cementoblastoma include condensing osteitis, osteoblastoma, odontoma, cemento-ossifying fibroma, periapical cemental dysplasia, and hypercementosis [1]. The preferred treatment for cementoblastoma involves excision of the tumor, extraction of the involved tooth, and osseous curettage. While apical resection and root canal treatment may serve as alternative treatments, they carry an increased risk of recurrence [1,2].

In this report, we present a rare case of giant cementoblastoma associated with multiple teeth (5 teeth in the lower left region), extending to the buccal and lingual areas, and causing perforation of the mandibular cortex.

## Case presentation

**Fig. 1:**
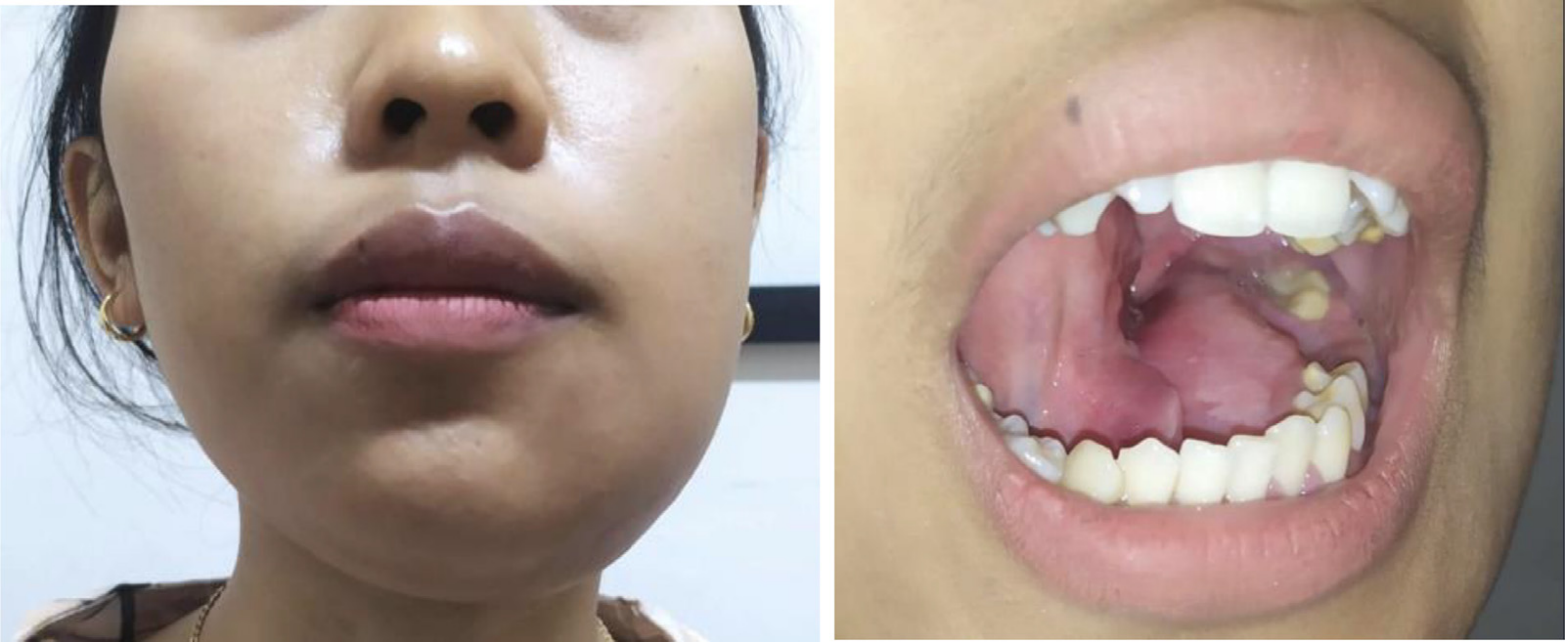
Clinical image shows intraoral swelling and facial asymmetry in the left lower region. There was no caries on the teeth involved.

A 19-year-old female patient presented to the Department of Oral and Maxillofacial Surgery with a 1-year history of pain, progressive swelling, and facial asymmetry in the left lower region. No maxillofacial trauma was reported. Upon extraoral examination, a diffuse swelling involving the left lower face was observed, which was tender and exhibited bony hardness upon palpation. Intraorally, there was swelling in the left mandibular gum area extending to the left buccal and lingual areas, accompanied by a sunken first molar and a half-sunken second molar. No caries was noted on the teeth in the left lower region (Fig. 1).

**Fig. 2:**
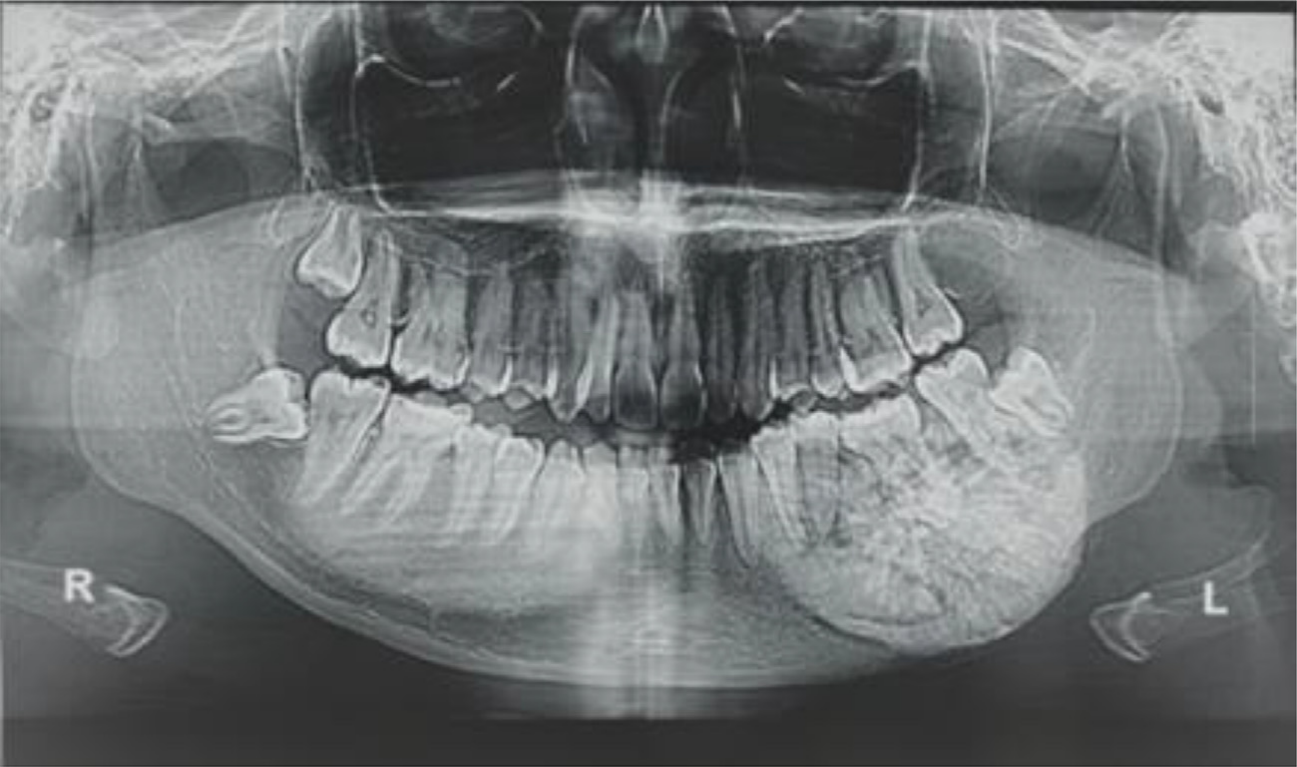
Panoramic radiograph demonstrating a radiopaque mass with well-defined borders, partially delineated by a thin radiolucent rim.

The initial panoramic radiograph revealed a giant, well-defined, relatively round, radiopaque mass partially delineated by a thin radiolucent halo. The mass was in contact with the roots of the first left lower premolar until the third impacted the left lower molar, involving a total of 5 teeth in the lower left region (Fig. 2).

**Fig. 3:**
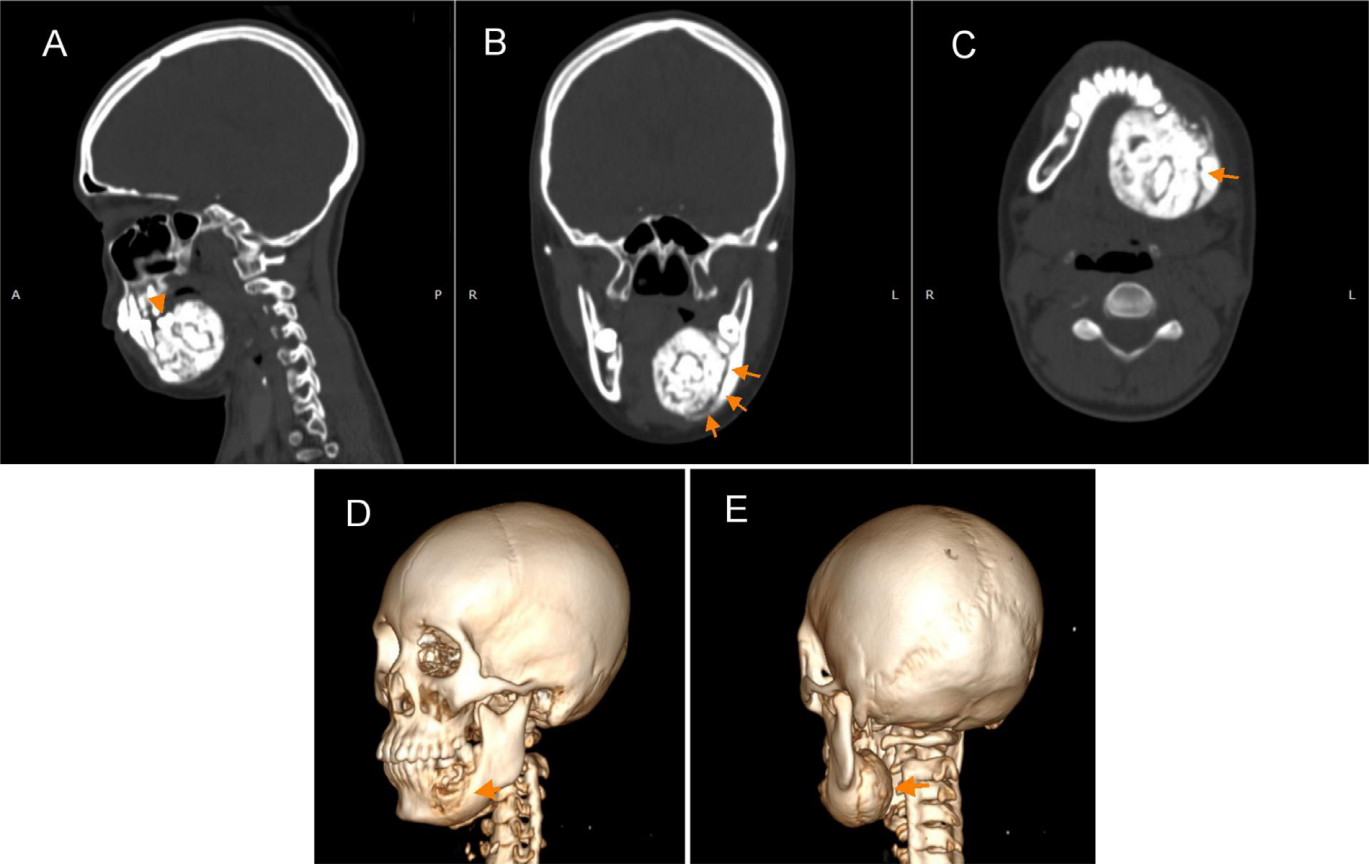
(A-C) Sagittal, coronal, and axial views of unenhanced head MSCT images illustrating a giant radiopaque mixed density mass in the posterior left region of the mandible with buccal and lingual extension and cortical perforation, partially surrounded by a thin radiolucent rim (arrow). In sagittal view (A), it shows the radiopaque mass fused to the root of the first molar of the left mandible (arrowhead). (D-E) Three-dimensional views depict cortical perforation of the mandible on the buccal and lingual sides caused by the enlarged mass (arrow).

Head MSCT demonstrated a radiopaque mixed-density mass measuring 4.40 × 4.02 × 4.75 cm, with significant buccal and lingual extension. Thinning of the adjacent buccal cortical bone and cortical perforation were observed. Oblique sagittal images revealed the mass's continuity with the root apex of the first molar, and a well-defined hypodense border between the mass and surrounding bone was evident (Fig. 3).

**Fig. 4:**
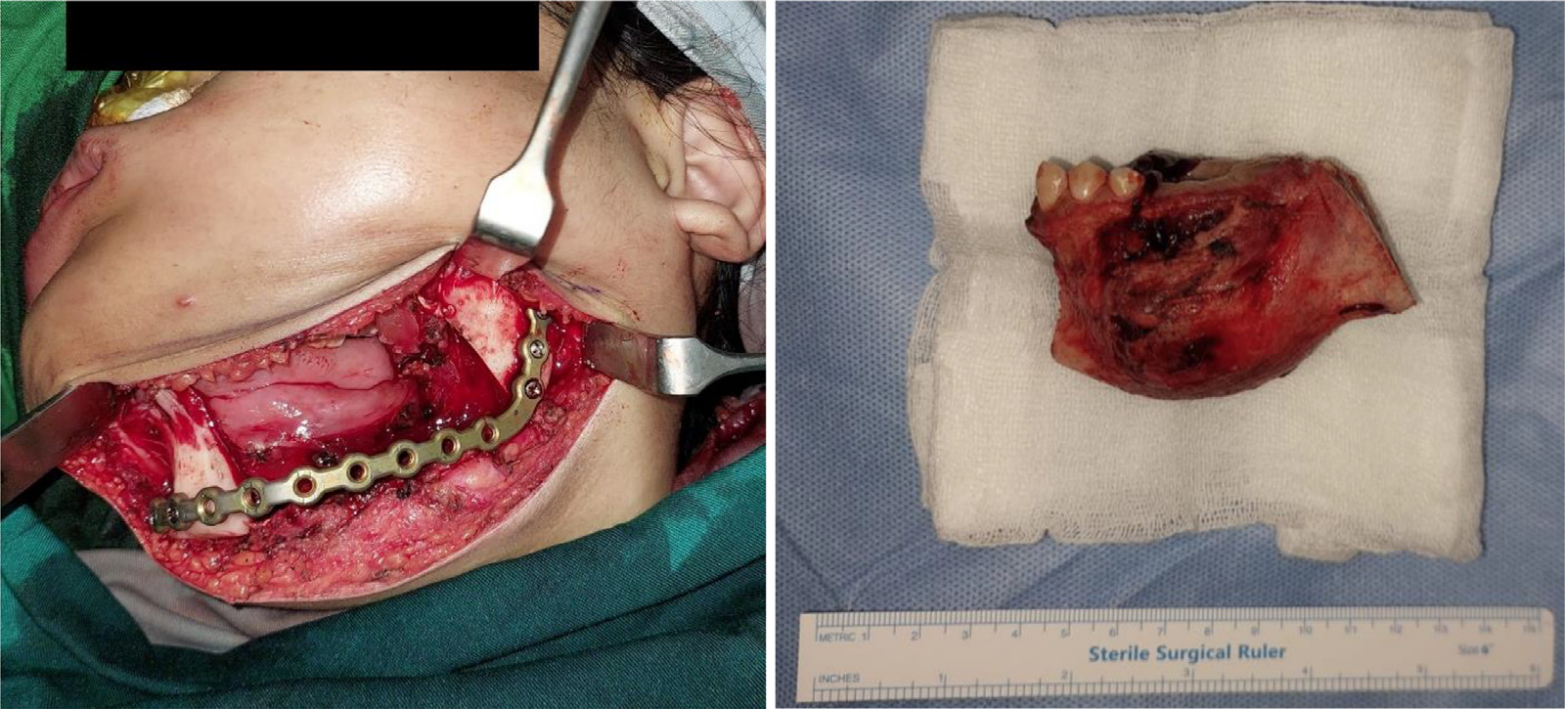
Intraoperative examination revealed segmental resection of the left mandible, with the tumor mass attached to the teeth structure and bone of the mandible. A Plate and screws were installed in place.

**Fig. 5:**
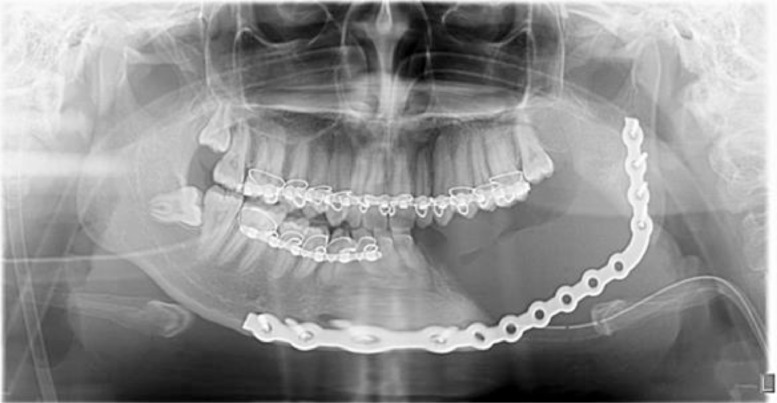
The panoramic radiograph illustrates the post hemimandibulectomy, with interdental wire, and a plate and screws installed in place. (1st day postoperative).

A biopsy was performed, confirming the mass as cementoblastoma. Based on the imaging and histopathology findings, the patient then underwent a left hemimandibulectomy due to the large and extensive nature of the mass to ensure complete removal (Fig. 4). Postoperative panoramic radiograph revealed no residual mass posthemimandibulectomy, and interdental wire, plate, and screw were installed in place (Fig. 5).

## Discussion

Cementoblastoma is an uncommon odontogenic tumor primarily affecting the posterior aspect of the mandible. The typical radiographic presentation involves a bone-density radiopaque lesion attached to the affected tooth's root, with a surrounding hypodense or radiolucent halo, distinguishing the lesion from the normal bone [1,2]. Cementoblastoma predominantly involves premolars or permanent first molars, with rare occurrences in the anterior mandible or maxilla, deciduous teeth, or association with multiple teeth [2,7]. The uniqueness of the present case lies in the exceptionally large cementoblastoma mass, involving multiple teeth (5 teeth in the lower left region), extending to the buccal and lingual areas, and causing significant destruction and perforation of the mandibular cortex.

When the lesion size is small in the early stages, cementoblastoma may remain asymptomatic. As the lesion progresses in size, patients may present with pain and swelling in the perioral region surrounding the implicated tooth, resulting in facial asymmetry. With further enlargement, the tumor may extend both buccally and lingually, causing erosion and potential destruction of the jaw bone cortex. This process can lead to displacement and/or involvement of adjacent teeth, ultimately progressing to invasion of the pulp and root canals [2,7]. In the current case, based on the multislice computed tomography (MSCT) images, it is evident that the tumor mass originated from the left first molar region and gradually expanded to involve a total of 5 surrounding teeth.

The characteristic radiologic manifestation of cementoblastoma typically presents as a radiopaque lesion directly attached to the tooth root, circumscribed by a radiolucent halo that demarcates it from the surrounding normal tissue [6,7]. In this particular patient, the mass exhibited mixed density on the MSCT image, primarily comprising a hyperdense mass resembling teeth. Notably, the radiolucent halo did not appear to encircle the entire mass. This observation can be attributed to the considerable size of the mass, resulting in the erosion of most of the mandibular cortex on the buccal and lingual aspects. Consequently, the demarcation between the mass and the normal mandibular bone tissue is no longer discernible in these regions.

**Fig. 6:**
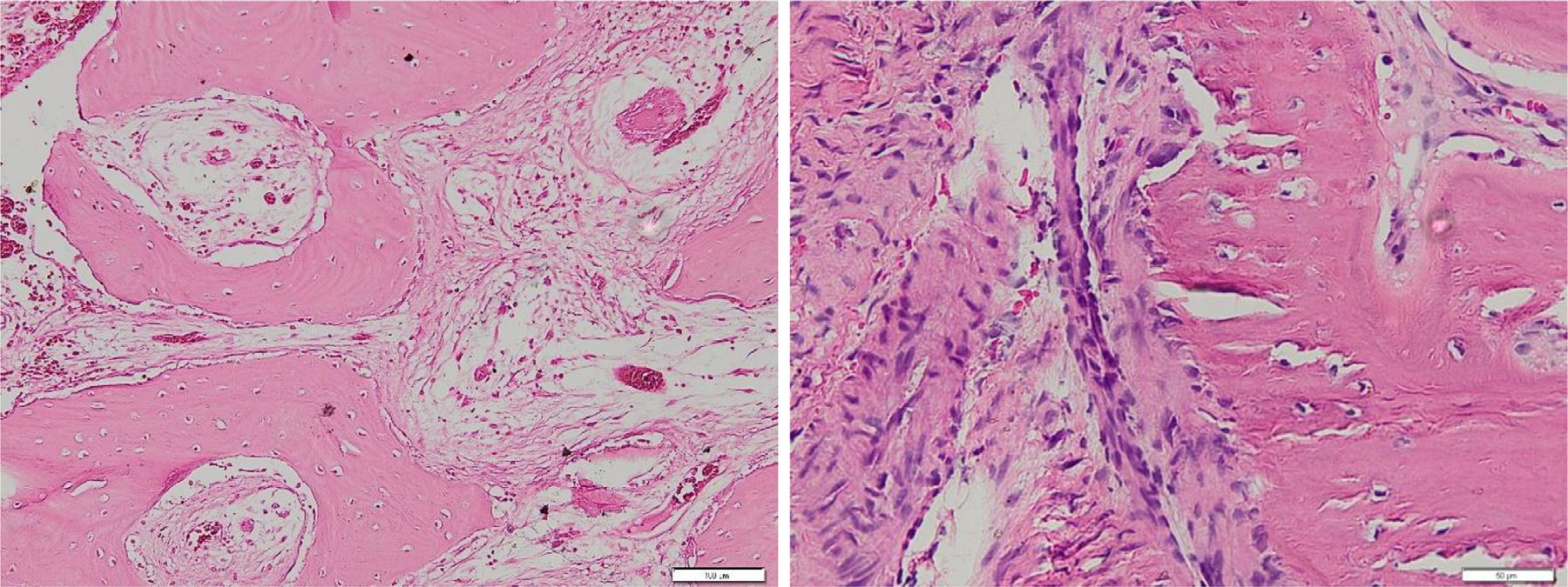
Histopathologic examination of hard tissue showed a trabecular arrangement of matrix cementum with cementoblast proliferation in between (H&E stain, ×100, ×200).

Cementoblastoma originates from the odontogenic ectomesenchyme of the cementoblast, which forms the lining of the tooth root [6,7]. Histopathologically, cementoblastoma manifests as a cementum-like layer forming a trabecular matrix with prominent basophilic reversal lines within its fibrovascular stroma. This presentation is accompanied by the proliferation of cementoblasts [4,^6^,7]. The more actively growing peripheral regions often lack mineralization (present as radiolucent rim on CT and conventional radiograph images) [4]. The histological features of cementoblastoma closely resemble those of osteoblastoma, atypical osteosarcoma, or osteoid osteoma [4]. However, a crucial point of differentiation lies in the connection of the cementum-like material layer to the tooth root, a characteristic feature exclusive to cementoblastoma [4]. In this case, the histopathologic findings were in concordance with the typical features of cementoblastoma (Figure 6).

**Fig. 7:**
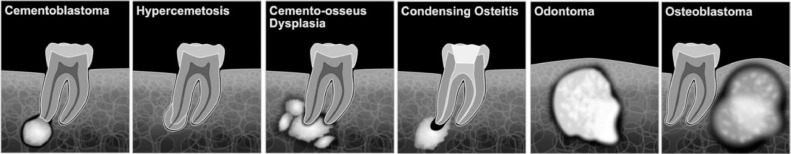
The illustration provided depicts cementoblastoma and various other radiopaque dentoalveolar lesions [8]. Reprinted with permission from Taghsimi K, Vasilyev AV, Kuznetsova VS, Galtsova AV, Badalyan VA, Babichenko II. The Efficiency and Safety of Dental Implantation in the Area of Hyperdense Jaw Lesions: A Narrative Review. Vol. 10, Dentistry Journal. MDPI; 2022.

Numerous radiopaque lesions within the dentoalveolar region may be considered potential differential diagnoses of cementoblastoma (Fig. 7) [8]. Differential diagnoses encompass condensing osteitis, osteoblastoma, odontoma, periapical cemento-osseous dysplasia, and hypercementosis [1]. Among these, osteoblastoma poses the greatest challenge for differentiation from cementoblastoma. The difficulty arises due to the striking similarity in radiologic and histopathologic appearances, particularly among males under 30 years of age [2,3]. Imaging examination should specifically focus on determining whether the radiopaque lesion is fused to the tooth root, a hallmark indicative of cementoblastoma. Additionally, osteoblastoma typically presents as a circumscribed lytic lesion without the presence of a radiolucent rim [2,^3^,6].

To aid in distinguishing among the numerous radiopaque lesions that constitute the differential diagnosis of cementoblastoma, the following table (Table 1) compares various periapical radiopaque lesions commonly observed, considering age, site predilection, clinical features, and radiologic characteristics. However, in some cases, the radiological appearance of these lesions is so similar that reliance on clinical history and histopathology data becomes imperative for establishing a more accurate diagnosis.

**Table 1:** Comparison of cementoblastoma with other periapical radiopaque lesions.

No	Diagnosis	Age	Site Predilection	Etiology	Clinical Features	Radiologic Features
1	Cementoblastoma	20-30 y.o Rare cases found in children involving primary teeth [9]	Mostly in the premolar or first molar of the mandible. Rare cases can be found in the maxilla [9].	Benign odontogenic neoplastic lesion	- Fused with the tooth root. - Arises from the cementoblasts in the PDL. - Associated with pain and bony expansion to the lingual/palatal aspect of the alveolar ridges. - Tooth is vital.	- Radiopaque periapical lesion fused to single/multiple teeth roots causes loss of root contour (root resorption). - Surrounded by a well-defined radiolucent periphery [10]. - Expansile, may cause the bony expansion to the lingual/palatal aspect of the alveolar ridges and cortical perforation. - No thickening of the PDL space.
2	Hypercementosis	20-30 y.o	Posterior area of the mandible	Non-neoplasm, excessive cementum deposition on the tooth root due to systemic and local factors [8]	- No clinical signs or symptoms. - Without pain or swelling and involves nearly the entire root area.	- Continuity of the periodontal ligament around the enlarged root, enveloping it as seen in normal cementum. - In rare cases, the enlarged root is significant enough to mimic cementoblastoma but without bony expansion or cortical perforation [11].
3	PCOD (Periapical Cemento-Osseous Dysplasia)	40-50 y.o mostly black women [12]	Mostly associated with one or more vital mandibular anterior teeth	Unknown- substitution of normal bone by fibrous tissue with mineralized structures	- Usually asymptomatic. Some lesions can be painful [12]. - Tooth is vital.	- Initially lytic then progress to a mixed-density lesion, often with a radiolucent rim. Later, the radiolucent rim becomes sclerotic, and in the mature stage, the lesion is radiopaque with poorly defined margins [12,13]. - The periodontal ligament is intact. - No fusion of the tooth root.
4	Condensing Osteitis	30-70 y.o	Mostly in the molar and premolar area of the mandible	Inflammatory stimulus from an inflamed dental pulp [14]	- Associated symptoms of infected teeth (carious dentin/pulpitis) [14]. - The tooth is often non-vital.	- Periapical, poorly marginated, nonexpansile, sclerotic lesion. - May be unifocal or multifocal [12]. - Absence of peripheral radiolucent rim and tooth-mass continuity. - The involved tooth usually has a thickened PDL space and/or periapical/pulpal inflammatory changes (e.g., granuloma, cyst, or abscess) [15].
5	Odontoma	10-20 y.o	Incisor and canine areas of the maxilla and mandible	Odontogenic tumor-like malformation (type of hamartoma) [16]	- Usually asymptomatic. - Found in routine radiographs as an incidental finding.or when assessing delayed tooth eruption [17].	- Typically pericoronal [18]. - It has a radiolucent rim-like cementoblastoma, but the mass in the capsule consists of rudimentary tooth-like deposits or an irregularly shaped deposit with opacity similar to teeth [19].
6	Osteoblastoma	20-30 y.o	Posterior area of the mandible	Benign non-odontogenic neoplastic lesion	- Not associated with tooth roots. - Arises in the medullary cavity [7]. - Have osteolytic borders and cause persistent pain [1].	- Absence of peripheral radiolucent rim and tooth-mass continuity. - More irregular mass radiopacity [1]. - A solitary, round, or oval radiolucent lesion with foci of radiopaque structures [20].

PDL: Periodontal ligament

Cementoblastoma is typically treated by tumor excision and extraction of the associated teeth. However, in this specific case, due to the considerable size of the mass and its perforation of the mandibular cortex, a hemimandibulectomy was performed. This involved the removal of half of the mandibular bone from the parasymphysis to the angulus, along with the associated teeth and tumor mass, ensuring complete elimination of the lesion. When completely excised without any residual mass, cementoblastoma exhibits a low recurrence rate. In instances of incomplete excision and removal, research indicates a recurrence rate of 37.1% in 44 reported cases [3,^6^,7]. The presence of bony expansion and cortical bone perforation further heightens the risk of recurrence in cases where removal is incomplete [21].

## Conclusion

Cementoblastoma is a benign tumor characterized by a low recurrence rate, despite its potential for unlimited growth (if complete excision and removal are achieved) [2]. The typical imaging feature is the fusion of the bony lesion with the tooth root, accompanied by a peripheral radiolucent or hypodense lesion. The optimal treatment approach for cementoblastoma involves total surgical excision of the tumor, as residual lesions may contribute to recurrence [3]. In early cases, there is a possibility of surgical conservation with the preservation of involved teeth. However, in advanced stages, it is advisable to remove both the tumor and the associated teeth to minimize the risk of recurrence [3,4].

## Patient consent

The patient has seen a version of the manuscript to be submitted/published and she gave her consent for her image or other information relating to her to be reported in the above-named manuscript for consideration of publication in the Radiology Case Reports (RCR).

The patient understands that protected health information such as identification number, billing information, and address, will not be published and that efforts will be made to conceal her identity. However, diagnostic or medical imaging may be published.

The patient understands that the material may be published in the Radiology Case Reports (RCR) Journal. As a result, she understands that the material may be seen by the general public. She understands that she may revoke consent at any time before publication, but once the information has been published revocation of the consent is no longer possible.
